# Advancements in the Genetics of Spontaneous Coronary Artery Dissection

**DOI:** 10.1007/s11886-023-01989-1

**Published:** 2023-11-18

**Authors:** Alexander E. Katz, Santhi K. Ganesh

**Affiliations:** 1 Department of Internal Medicine, Division of Cardiovascular Medicine, University of Michigan, Ann Arbor, USA; 2 Department of Human Genetics, University of Michigan, 7220, MSRB III, 1150 West Medical Center Drive, Ann Arbor, MI 48109-0644 USA

**Keywords:** Spontaneous coronary artery dissection, Genetics, Association studies, Complex genetics, Mendelian genetics, Systemic arteriopathy

## Abstract

**Purpose of Review:**

Spontaneous coronary artery dissection (SCAD) is a significant cause of acute myocardial infarction that is increasingly recognized in young and middle-aged women. The etiology of SCAD is likely multifactorial and may include the interaction of environmental and individual factors. Here, we summarize the current understanding of the genetic factors contributing to the development of SCAD.

**Recent Findings:**

The molecular findings underlying SCAD have been demonstrated to include a combination of rare DNA sequence variants with large effects, common variants contributing to a complex genetic architecture, and variants with intermediate impact. The genes associated with SCAD highlight the role of arterial cells and their extracellular matrix in the pathogenesis of the disease and shed light on the relationship between SCAD and other disorders, including fibromuscular dysplasia and connective tissue diseases.

**Summary:**

While up to 10% of affected individuals may harbor a rare variant with large effect, SCAD most often presents as a complex genetic condition. Analyses of larger and more diverse cohorts will continue to improve our understanding of risk susceptibility loci and will also enable consideration of the clinical utility of genetic testing strategies in the management of SCAD.

## Introduction

Spontaneous coronary artery dissection (SCAD) is recognized as a significant cause of acute myocardial infarction (AMI), particularly in young and middle-aged women. As implied by its name, SCAD represents a spontaneous separation of the medial layer of the coronary artery, and the typical clinical presentation includes chest pain and elevation of cardiac enzymes as to be expected with AMI [1–4]. The diagnosis of SCAD is most often made during coronary angiography, and in recent decades, the use of intracoronary imaging techniques such as optical coherence tomography (OCT) or intravascular ultrasound (IVUS) has facilitated increased recognition of the disease [5–7]. In addition to increased knowledge regarding the epidemiology and histopathology of SCAD, research in the last several years has begun to uncover the molecular underpinnings of SCAD and increased our understanding of the genetic basis of the disease [8, 9]. In this review, we provide an overview of the current state of knowledge of the genetic factors contributing to the development of SCAD and the clinical relevance of these findings.

**Fig. 1:**
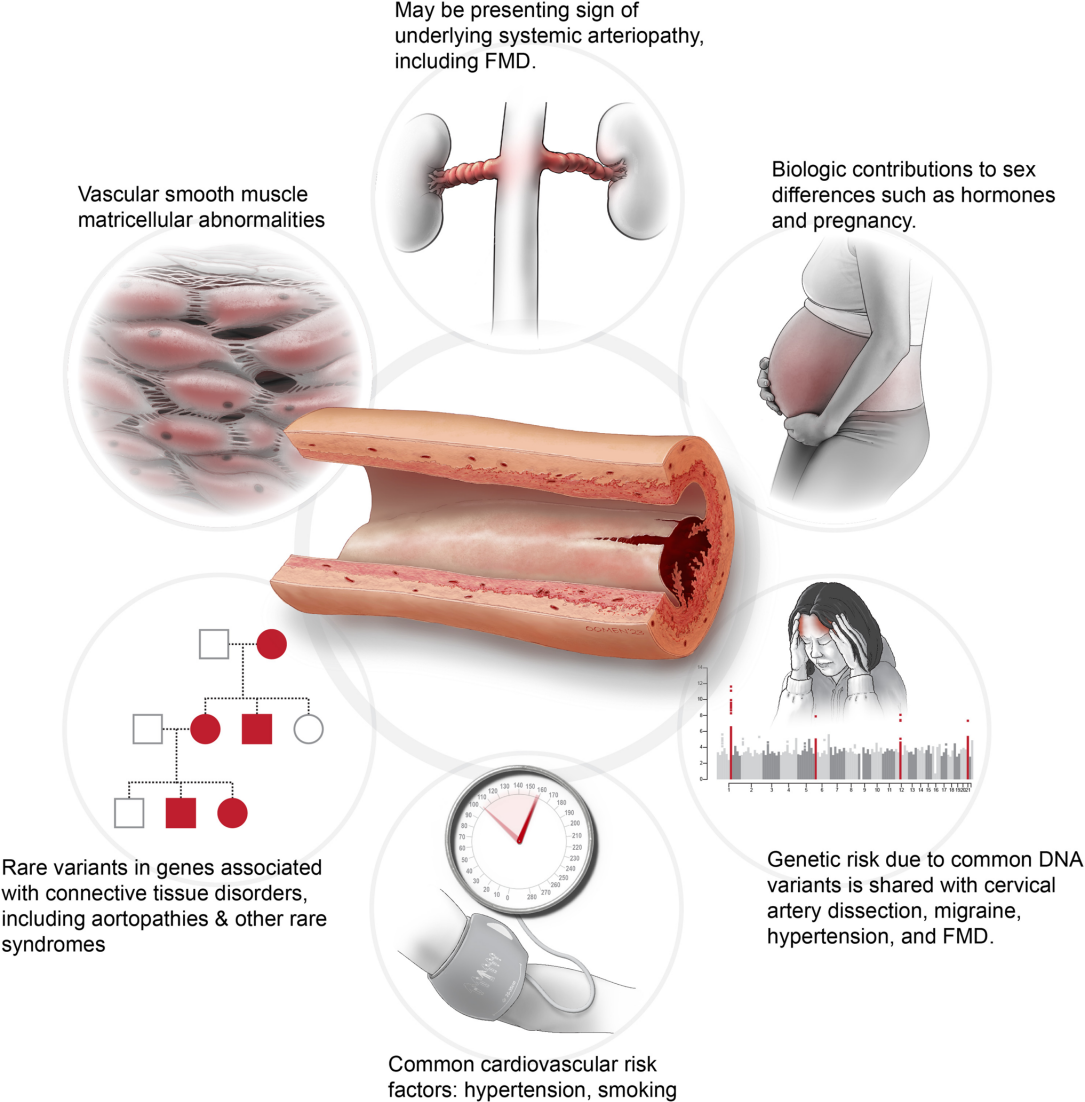
Genetic, biologic, and clinical associations to SCAD. SCAD is linked to several other conditions and may be indicative of an underlying systemic arteriopathy such as fibromuscular dysplasia (FMD, depicted as renal artery involvement in the top circle), with risk factors involving sex-specific biologic associations and hormonal milieu including pregnancy (right upper circle). SCAD has a complex genetic architecture that is also associated with FMD and migraine headache (right lower circle), with additional risks conferred by hypertension (lower circle) or other triggers. Monogenic connective tissue disorders have been implicated in a subset of individuals with SCAD (left lower circle), and genetic discovery studies thus far highlight biologic mechanisms involving matricellular alterations involving the arterial vascular smooth muscle (left upper circle)

While the underlying cause of SCAD is not completely understood and is likely multifactorial, more is known regarding the epidemiology, clinical features, and pathophysiology of the disorder. SCAD is most often diagnosed in women with fewer risk factors for atherosclerosis compared to the general population of individuals presenting with AMI [10, 11]. In women under the age of 50, SCAD is estimated to represent 22–35% of all cases of AMI [2, 4, 12]. Clinical factors that have been associated with SCAD (in addition to female sex) as potential triggers include hypertension, extreme exercise, emotional stress, and pregnancy-related considerations [2, 4, 13] although direct causal links have not been definitively demonstrated. SCAD has reliably been linked to extracoronary arteriopathies, most notably fibromuscular dysplasia (FMD) which is estimated to be a co-morbid condition in at least 50% of individuals presenting with SCAD [4, 14]. FMD is a nonatherosclerotic arteriopathy primarily affecting medium-sized arteries, with a similar strongly female-biased prevalence as SCAD, with variable arterial bed involvement and arterial involvement with stenotic disease, aneurysms, and dissections [14–17]. FMD is currently estimated to afflict 3.3% of the US population [18]. The high prevalence of additional extracoronary abnormalities in individuals presenting with SCAD is evidence that SCAD may be best thought of as an initial presentation of an underlying systemic arteriopathy [19], which has potential implications for clinical management and outcomes (Fig. 1).

**Fig. 2:**
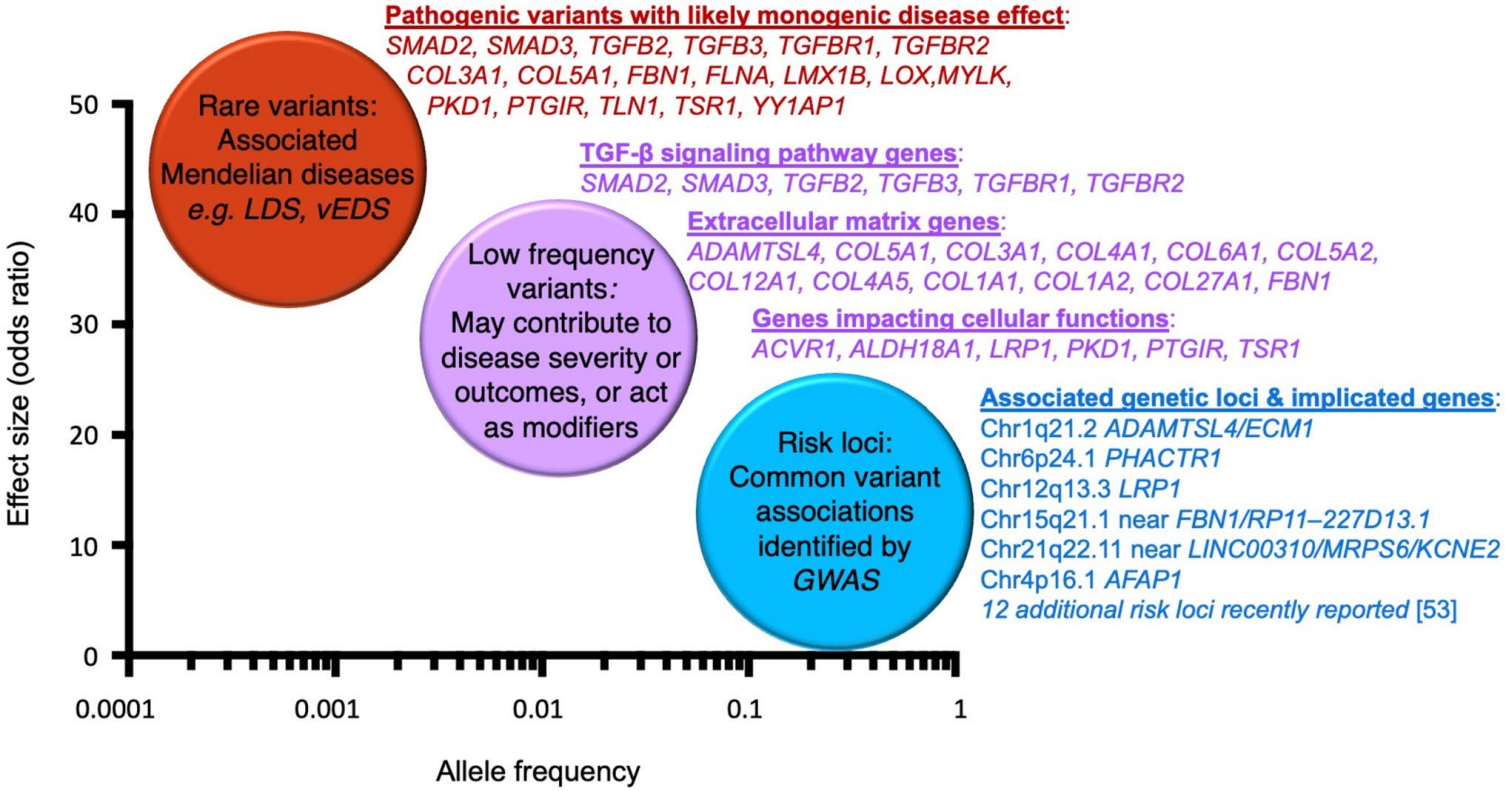
Emerging genetic architecture of SCAD. The genetic architecture of SCAD encompasses a spectrum of effect sizes and allele frequencies. Rare pathogenic variants in genes associated with Mendelian disorders confer large effect sizes. Low-frequency variants in some of the same genes and extracellular matrix genes would not meet clinical standards for pathogenicity for monogenic disease, but they have been demonstrated in aggregate to contribute to the phenotypic expression of SCAD. Common variants with lower effect sizes have also been found through association studies and represent risk susceptibility loci for SCAD. Effect size here is estimated through an inverse relationship with calculated or expected odds ratios. (Figure adapted from: Nature. 2009;461(7265):747–753, with permission of Springer Nature) [64]

The current hypothesis regarding an etiologic mechanism of SCAD is that the presence of an underling arteriopathy results in a weakened or more fragile coronary artery, and then a trigger in a susceptible individual leads to dissection, which is due to either an intimal tear or spontaneous hemorrhage between the layers of the wall, typically within the arterial tunica media [20]. While many aspects of the pathogenesis have yet to be determined, several recent advancements in the genetics of SCAD have identified both rare and common genetic factors that contribute to the underlying genomic architecture of the disease and provide insight into the relationship of SCAD with FMD in addition to other rare vascular syndromes and common cardiovascular diseases (Fig. 2).

## Rare Genetic Variants

In a minority of cases, individuals presenting with SCAD are found to harbor rare variants with strong evidence of pathogenicity in genes known to be associated with syndromic and non-syndromic forms of arterial disease. In such instances, SCAD may be thought of similarly to a Mendelian disorder, in which the effect of a variant in a single gene results in the development of disease, although with undefined penetrance for SCAD. Among a cohort of 179 people with SCAD who underwent targeted sequencing of 25 genes associated with thoracic aortic aneurysm and dissection (TAAD), 10.6% had variants annotated as deleterious by in silico predictors [21•]. These variants were formally classified as pathogenic (P), likely pathogenic (LP), and variant of uncertain significance (VUS) through the ACMG clinical standards and guidelines for genetic variant interpretation, with the majority annotated as VUS. Rare variants in *SMAD2* were significantly enriched in the SCAD cohort compared to the general population, as present in 0.8% of SCAD versus 0.1% of the gnomAD reference database. *SMAD2* was one of six genes associated with Loeys-Dietz syndrome (*TGFBR1*, *TGFBR2*, *SMAD2*, *SMAD3*, *TGFB2*, *TGFB3*) tested, and for which there was enrichment for rare variants in patients with SCAD when considered as a set of genes together; the other five genes did not meet statistical significance individually. In this cohort, rare variants with evidence of pathogenicity were also identified in *COL3A1* (associated with Vascular Ehlers-Danlos syndrome), *LOX* (familial thoracic aortic aneurysm and dissection), and *FLNA* (disorders associated with neuronal migration abnormalities) [21•]. None of the patients harboring P or LP variants in the above genes had the expected clinical presentations of the associated disorders (e.g., dilated aortic root in individuals with variants in Loeys-Dietz syndrome genes). This may reflect that there is wide phenotypic spectrum for these disorders, and SCAD may be the forme fruste, as the initial or only clinical manifestation for some people. To further investigate the shared Mendelian-effect genomic susceptibility between SCAD and vascular connective tissue disease genes underlying TAAD, the incidence of SCAD among all patients with TAAD has been evaluated. In a cohort of 7568 patients with known or suspected genetically mediated TAAD, only 11 (0.15%) were also found to have SCAD [22].

In a separate study, a cohort of 384 individuals with SCAD underwent exome sequencing in which all protein coding genes in the genome are evaluated, and 14 (3.6%) were found to have variants meeting clinical criteria for pathogenicity in a total of seven genes: *PKD1* (*n* = 5), *COL3A1* (*n* = 2), *SMAD3* (*n* = 2), *TGFB2* (*n* = 2), *LOX* (*n* = 1), *MYLK* (*n* = 1), and *YY1AP1* (*n* = 1) [23•]. Using 13,722 sequenced controls from UK Biobank, no genes from a candidate list (130 genes associated with arteriopathies in humans and 303 genes associated with phenotypes in mice) were found to have a clear genetic association meeting statistical threshold, with the strongest signal of enrichment found for variants in *PKD1* (identified in 1.7% of SCAD cases versus in 0.1% of controls, but not meeting study-wide significance). These findings are consistent with reports from clinical case series, in which individuals with SCAD undergoing clinical genetic testing are found to have variants in vascular connective tissue disease genes (including *COL3A1*) at a frequency of approximately 5% [24, 25].

Sequencing studies have also identified novel gene disease associations. Through a family-based sequencing approach, the cytoskeletal protein gene *TLN* has been associated with SCAD. In this analysis, a rare, missense variant in *TLN* was found to be segregating with disease in a family of three affected individuals, with 10 additional individuals in a cohort of 675 unrelated patients with SCAD also found to harbor rare missense variants in *TLN* [26]. *TLN* encodes for talin 1, a cellular cytoskeleton protein expressed in arterial tissue and endothelial cells [27], and alterations in the protein may affect the integrity of the arterial wall. A study of SCAD employing exome sequencing of 85 individuals with SCAD and 296 controls demonstrated an association with rare coding variants in *TSR1* [28]. In this cohort, *TSR1* was the strongest associated gene after applying a minor allele frequency cutoff of 1% and selecting for variants predicted to be deleterious through in silico analysis. *TSR1* encodes for a protein that is involved in the maturation of cellular ribosomes [29]. The enrichment for rare coding variants in *TSR1* was validated in a separate cohort of 53 affected individuals and 201 controls. Notably, this study included a lower percentage of women compared to that of the expected disease epidemiology (approximately 17% of the entire cohort were women) which may be because of the inclusion of patients with atherosclerotic disease-causing arterial dissection [28]. Additionally, this study was conducted in individuals of Han Chinese ancestry, whereas most other gene discovery studies of SCAD have comprised primarily European-ancestry individuals, and the demographics and clinical associations of SCAD have not been as well defined in non-European ancestry groups. In addition to the genes noted, the prostacyclin receptor gene *PTGIR* has been associated with FMD and rare, predicted loss-of-function (LOF) variants in *PTGIR* have been found in individuals with SCAD [30]. While no statistically significant enrichment for variants in *PTGIR* among individuals with SCAD has been identified, the potential involvement of prostacyclin signaling, which may influence vasodilation, vascular fibrosis, and platelet activation, warrants further investigation.

More recent analyses have suggested that in more general forms of SCAD (not necessarily those presenting for genetic evaluation in the clinic), the yield for pathogenic variants in vascular connective tissue disease genes may be higher than previously recognized. In a prospective, multi-center cohort of 336 individuals with SCAD, a subset of 94 individuals had SCAD with high-risk features defined as peripartum SCAD, recurrent SCAD, or in an individual with a family history of arterial dissection. In this subset hypothesized to harbor rare variants in candidate genes implicated in SCAD, rare variants in vascular connective tissue disease genes were found in 16 of 94 (17%) individuals, which were enriched compared to that of the gnomAD reference database [31••]. Among the genes queried, *COL3A1* and aggregated testing of Loeys-Dietz syndrome-associated genes (*TGFBR1*, *TGFBR2*, *SMAD2*, *SMAD3*, *TGFB2*, *TGFB3*) were the leading signals of enrichment, consistent with prior studies [21•, 24, 25]. In this cohort of individuals with SCAD with the defined high-risk features, there was additional enrichment for rare variants in genes prioritized by genome-wide association studies of SCAD, as well as genes prioritized by statistical analyses leveraging large databases of arterial tissue gene expression at the mRNA level, in the NIH genotype-tissue expression (GTEx) database, employing colocalization analysis (strongly prioritized genes were *ADAMTSL4*, *LRP1*, and *PHACTR1*) [32•]. Collectively, approximately 1/5 of individuals (20/94) with the high-risk SCAD phenotype had a rare variant identified, while only six variants would meet strict ACMG clinical criteria for pathogenicity. In a separate cohort of 91 unrelated sporadic SCAD cases, 10 individuals were found to have rare, likely disease-causing variants in a connective tissue disease gene [33]. Only one of these ten individuals had extracoronary manifestations consistent with a connective tissue disease. The yield of pathogenic or likely pathogenic variants of approximately 11% in this study is higher than in previously published reports. As variant annotation is expected to improve with additional research, the potential utility for clinical genetic testing for all individuals, versus a selected subset of individuals, with SCAD will need to be considered. All new gene findings, aside from those in genes previously known to be associated with vascular connective tissue diseases, will require further study to replicate and precisely define their biologic and clinical relevance.

## Complex Genetic Architecture Involving Common Variants

While rare genetic variants have been shown to play a role in some individuals, recent studies have demonstrated that common genetic variants also contribute to the complex genetic architecture of SCAD. The first single nucleotide polymorphism (SNP) found to be associated with SCAD was a non-coding variant in the *PHACTR1* gene (rs9349379-A) [34]. This variant was also the first known common genetic variant to be associated with FMD [35], but notably had also been previously shown to be associated with a wider range of phenotypes including migraine [36], hypertension [37, 38], and cervical artery dissection [39]. The same variant has also been associated with a reduced risk of more prevalent forms (e.g., atherosclerotic) of coronary artery disease and MI [40–42]. The SNP rs9349379 is located at chromosome 6p24.1, and follow-up studies (including integrative transcriptome and colocalization analysis) have demonstrated an association with the expression of the *PHACTR1* gene [32•]. *PHACTR1* encodes for a protein that is involved in the organization of the actin cytoskeleton, particularly in endothelial cells. There are at least five isoforms of the gene expressed in human coronary arteries, with rs9349379 specifically located at a MEF2 binding site and has been shown to be associated with *PHACTR1* transcript expression levels [43, 44]. Conflicting data from induced pluripotent stem cell modeling of the risk allele exists to prioritize *PHACTR1* at this genetic locus, with one study reporting an effect on *EDN1* gene expression [45] and another verifying the observation of effect regulation on the expression of *PHACTR1* [46]. Endothelin-1, encoded by *EDN1*, affects vasoconstriction and arterial remodeling which makes it a plausible candidate to be involved in disorders of arterial dysplasia. These observations highlight the challenges of identifying the causal genetic effect from GWAS.

Despite the challenges of conducting GWAS studies in diseases with relatively low prevalence, additional common genetic variants have been identified as associated with SCAD. A GWAS of women of European ancestry with SCAD (484 cases and 1477 controls in the discovery cohort, 183 cases and 340 controls in the replication cohort) identified associations with five loci (one of which was in the previously identified *PHACTR1* locus) [47•]. A 250-kb region at the chromosome 1q21.3 locus was identified to have the strongest association (OR, 1.78) with the authors reporting *ADAMTSL4*, *MRPS21*, and *ECM1* as the most likely genes mapping to this region. This was based on expression quantitative trait loci (eQTL) analyses from GTEx arterial tissue, which enables variants to be correlated with specific gene expression levels. A separate GWAS study of SCAD (analyzed in an initial discovery study with 270 cases and 5263 controls and replication study with 263 cases and 3207 controls) based upon a prospective, multi-center SCAD registry, found an association at chromosome 1q21.2 with rs12740679 influencing *ADAMTSL4* gene expression, and this gene was strongly prioritized through colocalization analysis (combining the GWAS result and an eQTL analysis of arterial tissue mRNA expression in GTEx). This locus had an OR of 1.8 (discovery cohort OR 1.97), demonstrating a consistent, relative strong effect for a common variant as compared to many GWAS signals for other traits and diseases [32•]. *ADAMTSL4* encodes a protein in the extracellular matrix that binds to fibrillin-1 to promote fibrillogenesis in the matrix [48]. Coding variants in *ADAMTSL4* have been associated with ectopia lentis, due to defective fibrillogenesis in the ocular zonules that suspend the lens of the eye. It is notable that fibrillin-1 is encoded by the *FBN1* gene, in which pathogenic variants cause Marfan syndrome and/or ectopia lentis, and SCAD has been described in individuals with Marfan syndrome [32•]. Additional analyses inclusive of meta-analysis of discovery and replication cohorts in this study also identified risk loci in *PHACTR1* at chromosome 6p24.1, in *LRP1* at chromosome 12q13.3, and in women only at chromosome 21q22.11 near *LINC00310*. *PHACTR1* and *LRP1* were strongly prioritized through the same GWAS-eQTL colocalization approach. *LRP1* encodes for low-density lipoprotein receptor-related protein 1 which is involved in several cellular processes, with additional genetic studies and mouse studies implicating *LRP1* in aortic aneurysms [49–51]. A polygenic risk score for SCAD was constructed from the GWAS result and found to be associated with a higher risk of SCAD in individuals with FMD; it was also associated with a higher risk of migraine headache and a lower risk of atherosclerotic coronary artery disease and MI in the Million Veteran Program and UK Biobank cohorts. The same polygenic score for SCAD was associated with abdominal aortic aneurysms in the Million Veteran Program [52]. These findings raise important questions regarding the underlying biologic mechanisms for the observed sex-biased prevalences of these diseases, with SCAD occurring in women in approximately 90% of cases whereas atherosclerotic coronary artery disease and abdominal aortic aneurysms both have notably higher prevalence in men.

More recently, a meta-analysis of GWAS studies collectively evaluating 1917 SCAD cases and 9292 controls of European ancestry has been performed. This study identified 12 novel risk loci in addition to the five previously identified loci [53••]. Prioritized genes identified in newly found risk loci, including *COL4A1*, *COL4A2*, *HTRA1*, and *TIMP317*, reaffirm the importance of the extracellular matrix to the pathophysiology of SCAD. A risk locus containing *F3*, a critical gene in the coagulation cascade, suggests that impairment in tissue-mediated coagulation may also contribute to the disease process. A locus containing *AFAP1* was also implicated in this study, which had previously been identified in a discovery analysis of pregnancy-associated SCAD [54] but had not been replicated or previously reported in SCAD overall. The study of pregnancy-associated SCAD (defined as SCAD occurring during pregnancy, following miscarriage, or within 1 year postpartum) included a discovery cohort of 53 cases and 1477 controls with a replication cohort of 32 cases and 334 controls which were part of the larger SCAD GWAS as well. This risk locus was notable for its high odds ratio (OR, 4.63) in the study of pregnancy-associated SCAD, with *AFAP1* as the prioritized positional candidate gene given its involvement in prolactin signaling [54]. Such recent efforts highlight how the accrual of larger cohorts can be expected add to our understanding of the genetic risk loci for SCAD.

## Contribution of Intermediate Effect Alleles to SCAD’s Genetic Architecture

Studies have also suggested that low-frequency alleles that do not have effect sizes strong enough to be considered pathogenic may nonetheless be contributing to the genetic architecture of SCAD. Several studies of SCAD have reported sequencing-defined genetic variants that were low-frequency, but not rare enough to be considered pathogenic by clinical testing standards, in the reference databases [21•, 23•, 31••, 55•, 56]. These studies have highlighted the role that fibrillar collagen genes, which serve vital functions in the formation of the extracellular matrix, may play in the pathogenesis of SCAD. A study involving 228 individuals with SCAD (130 in the discovery cohort and 98 in the replication cohort) implicated a set of 10 collagen genes (*COL3A1*, *COL5A1*, *COL4A1*, *COL6A1*, *COL5A2*, *COL12A1*, *COL4A5*, *COL1A1*, *COL1A2*, and *COL27A1*) as significantly enriched for rare, deleterious variants in aggregate compared to 46,559 UK Biobank controls, when analyzing a selected candidate set of genes based upon 2506 genes expressed in the coronary arteries in GTEx [55•]. Individuals with SCAD were 1.75 times more likely to harbor rare, deleterious variants in fibrillar collagen genes compared to UK Biobank controls. While these low-frequency variants can be considered disruptive, they would not be considered clinically pathogenic regarding potential Mendelian effect sizes. Rather, such variants are more appropriately thought of as intermediate effect alleles and potential modifiers of the underlying disease process, thereby increasing the risk of SCAD.

Further support to the notion that rare variants in fibrillar collagen genes can act as modifiers of the SCAD phenotype comes from an exome sequencing study involving a cohort of 264 individuals with multifocal FMD. This study identified a recurrent pathogenic variant in *COL5A1*, c.1540G>A, p.(Gly514Ser) in four unrelated probands with FMD, one of whom was included in the exome study. Among the entire cohort of individuals with FMD, a higher burden of rare and low-frequency variants predicted to be deleterious by in silico analysis (but of uncertain clinical significance, except for *COL5A1*, c.1540G>A) in *COL5A1* was found in individuals with arterial dissections, including of the coronary arteries, as manifestations of their disease [56]. Notably, the *COL5A1*, c.1540G>A variant, was in fact rare, with zero observations in the gnomAD reference database and was newly established as a variant annotated as a likely pathogenic variant that is located on a shared ancestral haplotype, supporting a "founder effect," the implication of which is that additional individuals harboring this specific variant are likely to exist in the population. SCAD was the presenting clinical manifestation in one of the four probands with this rare variant. The specific nature of how such variants in *COL5A1* (which is typically associated with classical Ehlers-Danlos syndrome) are contributing to spontaneous arterial dissections, including SCAD, requires further study. Cohorts with larger numbers of individuals with SCAD are expected to clarify the existing findings and potentially identify additional genes in which intermediate effect alleles may be functioning to increase SCAD risk as disease modifiers.

## Clinical Genetics Considerations for SCAD

Ultimately, our increased understanding of the genetics of SCAD has the promise to improve the clinical management of affected and at-risk individuals. Consensus guidelines have not been established for routine genetic testing of any individual with SCAD, but would be reasonable to consider for a subset of patients with high-risk disease features, such as recurrent SCAD [31••], pregnancy-related SCAD, or extensive dissection especially when involving the left main or proximal coronary arteries [22]. Following a SCAD event, head to pelvis angiographic imaging is generally recommended to evaluate for the presence of an underlying arteriopathy and additional arterial beds that may be affected. Imaging can help establish burden of disease and guide decisions about genetic testing [2, 19, 57].

For a patient who is found to have a pathogenic variant through clinical genetic testing, familial cascade testing should be offered to all first-degree relatives at a minimum (which necessitates adequate genetic counseling for the patient and family members). For the affected individual who has a pathogenic variant, additional screening and management should account for the phenotypic spectrum of disease associated with the specific gene. For example, a patient presenting with SCAD who is found to have a pathogenic variant in *COL3A1* should undergo a comprehensive evaluation for any additional signs and symptoms associated with vascular Ehlers-Danlos syndrome. Even without additional clinical manifestations, this individual would meet diagnostic criteria for vascular Ehlers-Danlos syndrome and should be managed according to best practice guidelines [58]. Similarly, any individual diagnosed with vascular Ehlers-Danlos syndrome should be aware of the potential for SCAD to be a manifestation of the disease, as is true of other Mendelian vascular disorders such as Loeys-Dietz syndrome. In the absence of an identifiable pathogenic variant, routine genetic screening of family members of individuals with SCAD is not recommended. Given the overlap between FMD and SCAD, a similar approach can be taken in SCAD as is currently recommended for FMD, in which any signs or symptoms of vascular insufficiency in a first-degree family member warrant further workup inclusive of arterial imaging [2, 9, 19, 57, 59].

As a complex genetic disorder, a polygenic risk score for SCAD has the potential to identify individuals at higher risk for disease. SCAD polygenic risk scores have been applied in a research context and have demonstrated shared genetic architecture between SCAD, FMD, and other vascular disorders such as arterial aneurysms [32•, 52]. In general, polygenic scores that are established in one ancestry group do not transport reliably to other ancestry groups [60, 61]. The apparent disparity in the current studies of SCAD, which have a strong European-ancestry bias, will need to be addressed to study and implement polygenic risk scores equitably. Ultimately, polygenic risk scores may be able to improve SCAD risk assessment, which may be particularly useful in at-risk populations such as family members of individuals with SCAD. Because of the complex genetic nature of SCAD encompassing both rare and common variants, it is conceivable that a polygenic risk score would explain the distinct phenotypes among family members who harbor the same rare genetic variant associated with SCAD. How polygenic risk scores for SCAD can be used in a clinical setting will be an area of future research, as is the case with polygenic risk scores for other complex cardiovascular disorders as well [61–63].

## Conclusions

As SCAD is now increasingly recognized as a significant cause of AMI, recent research efforts to enroll and study afflicted patients have improved our understanding of the molecular underpinnings of SCAD. The genetic basis of SCAD includes a wide spectrum of potential genetic mechanisms, including both rare and common genetic variation. While SCAD can occur as part of a classical Mendelian disease with a single, rare pathogenic variant conferring a large effect, it is more likely to present as a complex disorder attributable to multiple variants interacting with potential environmental factors or other triggers. Both rare and common variants have been shown to play a role in the complex genetic architecture predisposing an individual to SCAD. That SCAD can occur as part of a rare genetic syndrome (such as vascular Ehlers-Danlos syndrome) as well as part of more common vascular disorders such as FMD highlights the pleiotropic nature of the genetic risk. While impressive advancements in the genetics of SCAD have been made in recent years, larger cohorts with ancestrally diverse participants will be crucial to continue to uncover new mechanisms of disease and generate a more complete picture of the genomic factors predisposing individuals to SCAD. The translation of these genetic findings holds promise for contributing to clinical diagnostic utility and risk stratification, and also to the development of mechanistically targeted therapies based upon better understanding of the biology of SCAD.
